# Identifying the key characteristics, trends, and seasonality of pedestrian traffic injury at a major trauma center in Saudi Arabia: a registry-based retrospective cohort study, 2017–2022

**DOI:** 10.1186/s12873-024-01051-5

**Published:** 2024-07-29

**Authors:** Rayan Jafnan Alharbi, Abdulrhman Saleh Alghamdi, Rami Al-Jafar, Ateeq Almuwallad, Sharfuddin Chowdhury

**Affiliations:** 1 Department of Emergency Medical Services, College of Applied Medical Sciences, Jazan University, Al Maarefah Rd, Jazan, 45142 Saudi Arabia; 2 Emergency Medical Services Department, College of Applied Medical Sciences, King Saud Bin Abdulaziz University for Health Sciences, Riyadh, Saudi Arabia; 3 King Abdullah International Medical Research Center, Riyadh, Saudi Arabia; 4 Data Services Sector, Lean for Business Services, Riyadh, Saudi Arabia; 5 School of Public Health, Imperial College London, London, UK; 6 Trauma Center, King Saud Medical City, Riyadh, Saudi Arabia

**Keywords:** Pedestrians, Motor vehicles, Accidents, Wounds and injuries, Trauma centers, Public health, Saudi Arabia

## Abstract

**Background:**

Pedestrian traffic injuries are a rising public health concern worldwide. In rapidly urbanizing countries like Saudi Arabia, these injuries account for a considerable proportion of trauma cases and represent a challenge for healthcare systems. The study aims to analyze the key characteristics, seasonality, and outcomes of pedestrian traffic injuries in Riyadh, Saudi Arabia.

**Methods:**

This study was a retrospective cohort analysis of all pedestrian traffic injuries presented to King Saud Medical City, Riyadh, and included in the Saudi Trauma Registry (STAR) database between August 1, 2017, and December 31, 2022. The analysis of metric and nominal variables was reported as mean (standard deviation, SD) or median (interquartile range, IQR) and frequencies (%), respectively. A logistic regression analysis was performed to examine the influence of patients’ pre-hospital vitals and key characteristics on arrival at the ED on the need for mechanical ventilation and in-hospital mortality.

**Results:**

During the study period, 1062 pedestrian-injured patients were included in the analysis, mostly males (89.45%) with a mean (SD) age of 33.44 (17.92) years. One-third (35.88%) of the patients were Saudi nationals. Two-thirds (67.04%) of the injuries occurred from 6 p.m. until 6 a.m. Compared to other years, a smaller % of injury events (13.28%) were noticed during the COVID-19 pandemic (2020). Half (50.19%) of the patients were transported to the emergency department by the Red Crescent ambulance, and 19.68% required intubation and mechanical ventilation. Most of the patients (87.85%) were discharged home after completion of treatment, and our cohort had a 4.89% overall mortality. The logistic regression analysis showed the influence of patients’ pre-hospital vitals and key characteristics on arrival at the ED on the need for mechanical ventilation (Chi^2^ = 161.95, *p* < 0.001) and in-hospital mortality (Chi^2^ = 63.78, *p* < 0.001) as a whole significant.

**Conclusion:**

This study details the demographic, temporal, and clinical trends of pedestrian traffic injuries at a major Saudi trauma center. Identifying high-risk individuals and injury timing is crucial for resource allocation, targeting road safety interventions like public awareness campaigns and regulatory reforms, and improving prehospital care and patient outcomes.

## Introduction

Road traffic injuries remain an alarming and persistent global health challenge, resulting in substantial morbidity and mortality. The World Health Organization (WHO) estimates that road traffic crashes (RTCs) account for nearly 1.35 million deaths annually, with pedestrians forming a considerable fraction of this mortality [1]. The ramifications extend beyond the immediate loss of life, including long-term disabilities, significant healthcare costs, and a considerable emotional toll on families and communities [1]. The issue is particularly urgent in rapidly urbanizing nations where motorization and pedestrian injuries are sharply escalating [2]. Studies conducted in diverse geographies, such as China, Chile, and North Carolina, have shown common factors that significantly impact pedestrian injuries. Among these factors, age, time of the day, and location have been recurrent themes [3–5]. For instance, older adults and children appear more susceptible to severe injuries [6], and most pedestrian incidents occur during the evening and in urban settings [6].

Saudi Arabia presents a unique case study within this broader global context. The country has one of the highest rates of RTCs per capita globally [7, 8]. Within this setting, pedestrians comprise a notably vulnerable demographic, contributing significantly to fatalities and injuries [9]. Previous research has established general trends in Saudi Arabia’s road safety landscape, highlighting issues like high-speed driving, lack of adherence to traffic laws, and an increasingly complex road network due to rapid urbanization [9]. However, there needs to be more literature on pedestrian injuries in Saudi Arabia. Although early research did emphasize high fatality rates among pedestrians, attributing them to factors such as speeding, failing to yield right-of-way, and jaywalking [10], more recent studies have adopted a nuanced approach. These analyses have focused on micro-level factors like the efficacy of crosswalks, signal timings, and the role of public awareness campaigns in improving pedestrian safety [10].

Several hospital-based studies have provided data on the clinical aspects of pedestrian injuries in Saudi Arabia. These studies show high rates of severe injuries that necessitate surgical intervention and prolonged hospital stays, thereby increasing the burden on the healthcare system [11]. Furthermore, demographic patterns have emerged in the data. Studies reveal that young males, in particular, have a higher propensity for pedestrian injuries, aligning with global trends but also presenting a distinct challenge for public health policymakers in Saudi Arabia [12, 13].

The study aimed to identify the key characteristics, trends, and seasonality of pedestrian traffic injury presented at King Saud Medical City (KSMC), a major trauma center in Saudi Arabia. The secondary aim was to examine the influence of patients’ pre-hospital vitals and key characteristics on arrival at the ED on the need for mechanical ventilation and in-hospital mortality.

## Methods

Setting: King Saud Medical City (KSMC), a tertiary care government hospital under the Ministry of Health and a designated level-1 trauma center in Riyadh, Saudi Arabia. More than 1,400 beds are available at KSMC, making it one of the largest hospitals in Saudi Arabia. Of these, 100 are emergency department (ED) beds, and 200 are intensive care unit (ICU) beds. Annually, over two thousand trauma patients are admitted to KSMC [14]. The Saudi TraumA Registry (STAR) is an electronic database developed in 2017 at KSMC. It is based only on trauma patients admitted to the KSMC, with a vision to expand regionally and nationally in the near future. Under the directorate of the trauma center, the trauma registry department employs six data collectors qualified by the Association for the Advancement of Automotive Medicine (AAAM). The registry includes all patients who have a main diagnosis of injury and meet one of the following criteria: hospitalization lasting at least three calendar days, death in the emergency department or during inpatient stay following the injury, or any admission to the Intensive Care Unit (ICU) after injury. The registry collects information on the trauma patient’s journey from prehospital to discharge, encompassing 83 variables [13].

**Fig. 1:**
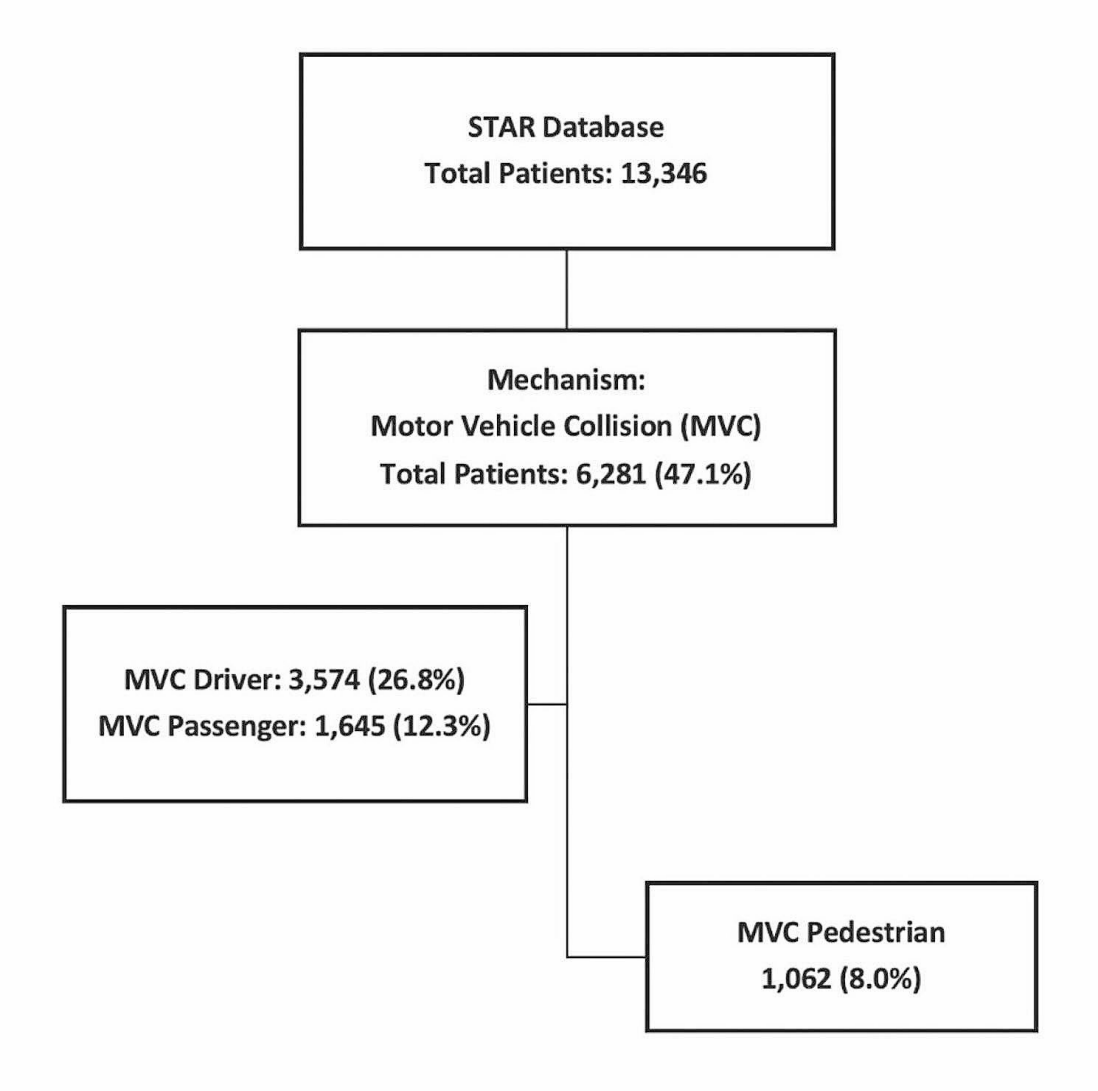
Selection of patients

Design and Data: This study was a retrospective cohort analysis of all pedestrian traffic injuries presented to KSMC and included in the STAR database between August 1, 2017, and December 31, 2022 (Fig. 1).

Data related to demographics (age, gender, nationality), injury event, prehospital vitals, procedures at the scene, mode of arrival at ED, respiratory assistance, trauma team activation, vitals and other parameters on arrival to ED, injury type, injury severity score (ISS), blood transfusion at ED, procedures in the ED, mechanical ventilation, operative procedure, disposition from ED, hospital and ICU length of stay, discharge destination from ED, complications, and mortality were collected for analysis. The records were cleaned to optimize the quality of the analyses.

Statistical analysis: Analysis of metric variables such as age, prehospital systolic blood pressure (SBP), heart rate (HR), respiratory rate (RR), on arrival at the ED, SBP, HR, RR, oxygen saturation, body temperature (^0^C), Glasgow coma scale (GCS), blood P^H^, base excess, International normalized ratio (INR), ISS, length of stays in days in intensive care unit (ICU) and hospital were reported as mean (standard deviation, SD), and median (interquartile range, IQR).

Analysis of nominal variables such as gender, nationality, injury event, procedures at the scene, mode of hospital arrival, injury type, respiratory assistance, trauma team activation, blood transfusion at the ED, procedures in the ED, operative procedure, mechanical ventilation, disposition from the ED, discharge destination from the hospital, in-hospital complications, and mortality were reported as frequencies (%). For seasonality, the trauma events were categorized year by year and plotted as a line graph.

A logistic regression analysis was performed to examine the influence of age, male gender, prehospital SBP, prehospital HR, prehospital RR, on arrival at ED, the SBP, HR, RR, SPO_2_, body temperature, GCS, blood P^H^, base excess, INR, blood transfusion, and ISS on variable mechanical ventilation (dependent) to predict the value “No.”

Another logistic regression analysis was performed to examine the influence of age, male gender, prehospital SBP, prehospital HR, prehospital RR, on arrival at the ED, the SBP, HR, RR, SPO_2_, body temperature, GCS, blood P^H^, base excess, INR, Blood transfusion in the ED, mechanical ventilation, and ISS on variable mortality (dependent) to predict the value “No.” A *p*-value of < 0.05 was considered significant.

The analysis was carried out by DATAtab Team (2024). DATAtab: Online Statistics Calculator. DATAtab e.U. Graz, Austria. URL https://datatab.net.

The study received approval from the KSMC institutional review board (IRB) (ref. H1RI-12-June23-01). The IRB committee granted a waiver from the obligation to obtain informed consent from the participants for the retrospective analysis of registry data.

## Results

During the study period, 1062 pedestrian-injured patients were included in the analysis, primarily males (89.45%) (Table 1) with a mean (SD) age of 33.44 (17.92) years (Table 2). One-third (35.88%) of the patients were Saudi nationals, and the rest were non-Saudis, predominantly from Bangladesh (11.49%), Yemen (8.47%), Pakistan (7.91%), India (7.34%), Sudan (6.59%), and Egypt (5.37%) (Table 1).

**Table 1:** Nominal variables: demographics (gender, nationality), injury event and other characteristics

Characteristics	Frequency, n (%)
**Gender** (***n* = 1062)**	
Male	950 (89.45%)
Female	112 (10.55%)
**Nationality** (***n* = 1062)**	
Saudi	381 (35.88%)
Non-Saudi	681 (64.12%)
Bangladesh	122 (11.49%)
Yemen	90 (8.47%)
Pakistan	84 (7.91%)
India	78 (7.34%)
Sudan	70 (6.59%)
Egypt	57 (5.37%)
Syria	43 (4.05%)
Nepal	13 (1.22%)
Eritrea	12 (1.13%)
Ethiopia	10 (0.94%)
Philippines	7 (0.66%)
Other	36 (3.39%)
Unknown or not documented	59 (5.56%)
**Injury event, time of the day** (***n* = 1062)**	
06:01–18:00	350 (32.96%)
18:01–06:00	712 (67.04%)
**Injury event, year** (***n* = 1062)**	
2017 (Aug-Dec)	91 (8.57%)
2018	238 (22.41%)
2019	219 (20.62%)
2020	141 (13.28%)
2021	188 (17.70%)
2022	185 (17.42%)
**Injury event, quarterly** (***n* = 1062)**	
Jan-Mar	287 (27.02%)
Apr-Jun	247 (23.26%)
Jul-Sep	249 (23.45%)
Oct-Dec	279 (26.27%)
**Procedures at scene** (***n* = 1062)**	
Yes	405 (38.13%)
No	657 (61.87%)
**Mode of arrival at ED** (***n* = 1062)**	
Red Crescent ambulance	533 (50.19%)
Government ambulance	299 (28.15%)
Private ambulance	19 (1.79%)
Private vehicle	129 (12.15%)
Police vehicle	3 (0.28%)
Helicopter	6 (0.56%)
Walk in	4 (0.38%)
Unknown or not documented	69 (6.50%)
**Injury Type** (*n* = 1816)*	
Head and face	388 (21.37%)
Thorax	207 (11.40%)
Abdomen and pelvis	289 (15.91%)
Spine	246 (13.55%)
Upper extremities	188 (10.35%)
Lower extremities	498 (27.42%)
**Respiratory assistance** (***n* = 1062)**	
Unassisted respiration	796 (74.95%)
Assisted respiration	239 (22.51%)
Unknown or not documented	27 (2.54%)
**Trauma team activation** (***n* = 1062)**	
Yes	187 (17.61%)
No	875 (82.39%)
**Blood transfusion in ED** (***n* = 1062)**	
Yes	94 (8.85%)
No	968 (91.15%)
**Procedures in ED** (***n* = 1062)**	
Yes	767 (72.22%)
No	295 (27.73%)
**Operative procedure** (***n* = 1062)**	
Yes	696 (65.54%)
No	366 (34.46%)
**Mechanical ventilation** (***n* = 1062)**	
Yes	209 (19.68%)
No	853 (80.32%)
**Disposition from ED** (***n* = 1062)**	
General Ward	786 (74.01%)
ICU	193 (18.17%)
Operating theatre	74 (6.97%)
Died in ED	5 (0.47%)
Unknown or not documented	4 (0.38%)
**Discharge destination from hospital** (***n* = 1062)**	
Home	933 (87.85%)
Died/mortuary (including death in ED)	52 (4.89%)
Discharged against medical advice (DAMA)	31 (2.92%)
Transferred to another hospital	17 (1.60%)
Transferred to rehabilitation center	16 (1.51%)
Abscoded	9 (0.85%)
Not documented	4 (0.38%)
**Complications** (***n* = 1062)**	
No	1043 (98.21%)
Yes	19 (1.79%)
Wound infection	12 (1.13%)
Pulmonary embolism	3 (0.28%)
Deep vein thrombosis	2 (0.19%)
Unplanned return to theatre	2 (0.19%)
**Mortality (total)**	52(4.89%)

*The total number is higher than the patient number due to polytrauma; the same patient had traumas in different body areas

**Fig. 2:**
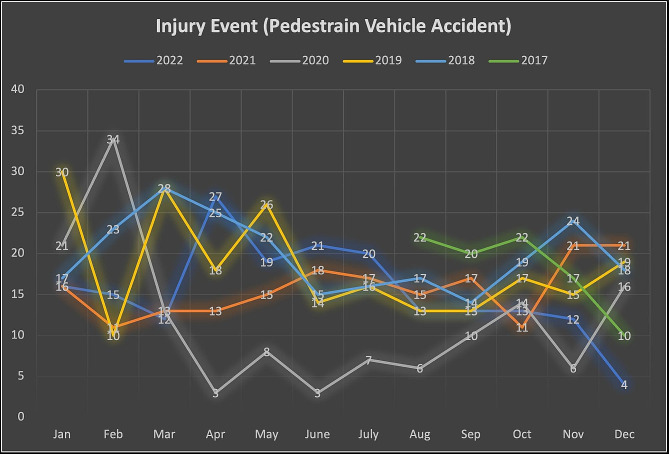
The line graph shows the trend of injury events month by month for the year (Aug 2017–2022)

Two-thirds (67.04%) of the injuries occurred from 6 p.m. until 6 a.m. During the summer months of April to September, there was a mild decrease (27–23%) in injury events. Compared to other years, a smaller percentage of injury events (13.28%) were noticed during the COVID-19 pandemic year 2020 (Table 1; Fig. 2).

The mode of transport to arrival at the ED was mainly by Red Crescent ambulance (50.19%), followed by government ambulance (28.15%) and private vehicle (12.15%). About three-fourths of the patients (74.95%) did not require respiratory assistance during transport. Lower extremities injury (27.42%) was the commonest injury type, followed by head injury (21.37%), abdomen, and pelvis (15.91%). On arrival at the ED, the trauma team was activated for 17.61% of patients. Only 8.85% of patients received a blood transfusion in the ED, and 19.68% required intubation and mechanical ventilation. Most of the patients were admitted to the general ward (74.01%), followed by admission to the ICU (18.17%). Most of the patients were discharged home (87.85%) after completion of treatment. Only 1.79% of patients developed complications during hospital stays, and our cohort had a 4.89% overall mortality (Table 1).

The mean (SD) prehospital SBP (mm Hg), HR (beat/min), and RR (rate/min) were 125.84 (21.31), 91.49 (17.28), and 17.48 (3.11) respectively. The mean (SD) GCS was 13.71 (3.08), ISS was 12.62 (9.31), hospital stays 15.23 (20.27) days, and ICU stays 13.23 (12.63) days. Table 2 tabulates the prehospital vitals and characteristics on arrival to the ED.

**Table 2:** Metric variables: demographics (age), prehospital vitals, characteristics on arrival at ED, and ICU and hospital length of stays

Variables	Mean (SD)	Median (IQR)	95% CI for mean
**Age in years**	33.44 (17.92)	31 (23)	32.36–34.52
**Prehospital**:			
SBP (mmHg)	125.84 (21.31)	124 (25)	123.90-127.77
HR (beat/min)	91.49 (17.28)	90 (21)	89.94–93.04
RR (rate/min)	17.48 (3.11)	18 (3)	17.20-17.77
**On arrival at ED**:			
SBP (mmHg)	126.19 (22.57)	125 (27)	124.80-127.57
HR (beat/min)	94.98 (20.48)	91 (24)	93.74–96.21
RR (rate/min)	20.18 (2.96)	20 (2)	20.00-20.36
SPO_2_	97.30 (2.89)	98 (3)	97.12–97.47
Body temperature (^0^C)	36.81 (0.37)	36.90 (0.30)	36.78–36.83
GCS	13.71 (3.08)	15 (0)	13.51–13.9
Blood P^H^	7.35 (0.09)	7.36 (0.08)	7.34–7.35
Base excess	3.52 (2.75)	3 (4)	3.33–3.70
INR	1.13 (0.33)	1.1 (0.20)	1.10–1.15
ISS	12.62 (9.31)	9 (12)	12.06–13.19
Days in hospital	15.23 (20.27)	9 (12)	14.02–16.45
Days in ICU	13.23 (12.63)	10 (13.50)	11.71–14.75

CI: Confidence intervals

The logistic regression analysis to examine the influence of age, male gender, prehospital vitals, and characteristics on arrival at ED and ISS on variable mechanical ventilation (dependent) showed that the model as a whole was significant (Chi^2^ = 161.95, *p* < 0.001). In particular, lower systolic BP (*p* = 0.002), lower GCS (*p* = < 0.001), patients who received a blood transfusion in ED (*p* = 0.02), and patients with higher ISS (*p* = 0.045) were significantly associated with needing definitive airway; mechanical ventilation (Table 3).

**Table 3:** Logistic regression model to examine the influence of age, gender, pre-hospital vitals, and characteristics on arrival at ED on mechanical ventilation

	Coefficient B	Standard error	z	*p*	Odds Ratio	95% CI
**Constant**	-11.91	42.68	0.28	0.78	0	0–1.45
**Age**	-0.04	0.02	1.91	0.056	0.96	0.92–1
**Male gender**	-3.42	2.69	1.27	0.204	0.03	0–6.39
**Prehospital**						
SBP (mm Hg)	-0.02	0.02	0.91	0.364	0.98	0.95–1.02
HR (beats/min)	-0.01	0.02	0.6	0.549	0.99	0.95–1.02
RR (rate/min)	-0.18	0.11	1.63	0.103	0.84	0.67–1.04
**On arrival at ED**						
SBP (mm Hg)	0.05	0.01	3.1	0.002*	1.05	1.02–1.08
HR (beats/min)	0	0.02	0.02	0.981	1	0.96–1.04
RR (rates/min)	0.01	0.13	0.05	0.958	1.01	0.78–1.30
SPO2	0.11	0.11	1.09	0.278	1.12	0.91–1.38
Body temperature (^0^C)	0.77	0.71	1.1	0.273	2.17	0.54–8.63
GCS	1.04	0.19	5.45	< .001*	2.83	1.95–4.12
Blood P^H^	-4.61	5.31	0.87	0.386	0.01	0–332.09
Base excess	-0.1	0.14	0.77	0.443	0.9	0.69–1.18
INR	0.72	2.47	0.29	0.77	2.06	0.02–261.66
Blood transfusion	-2.04	0.88	2.32	0.020*	0.13	0.02–0.73
ISS	-0.07	0.03	2	0.045*	0.94	0.88–1

*Statistically significant *p*-value

**Fig. 3:**
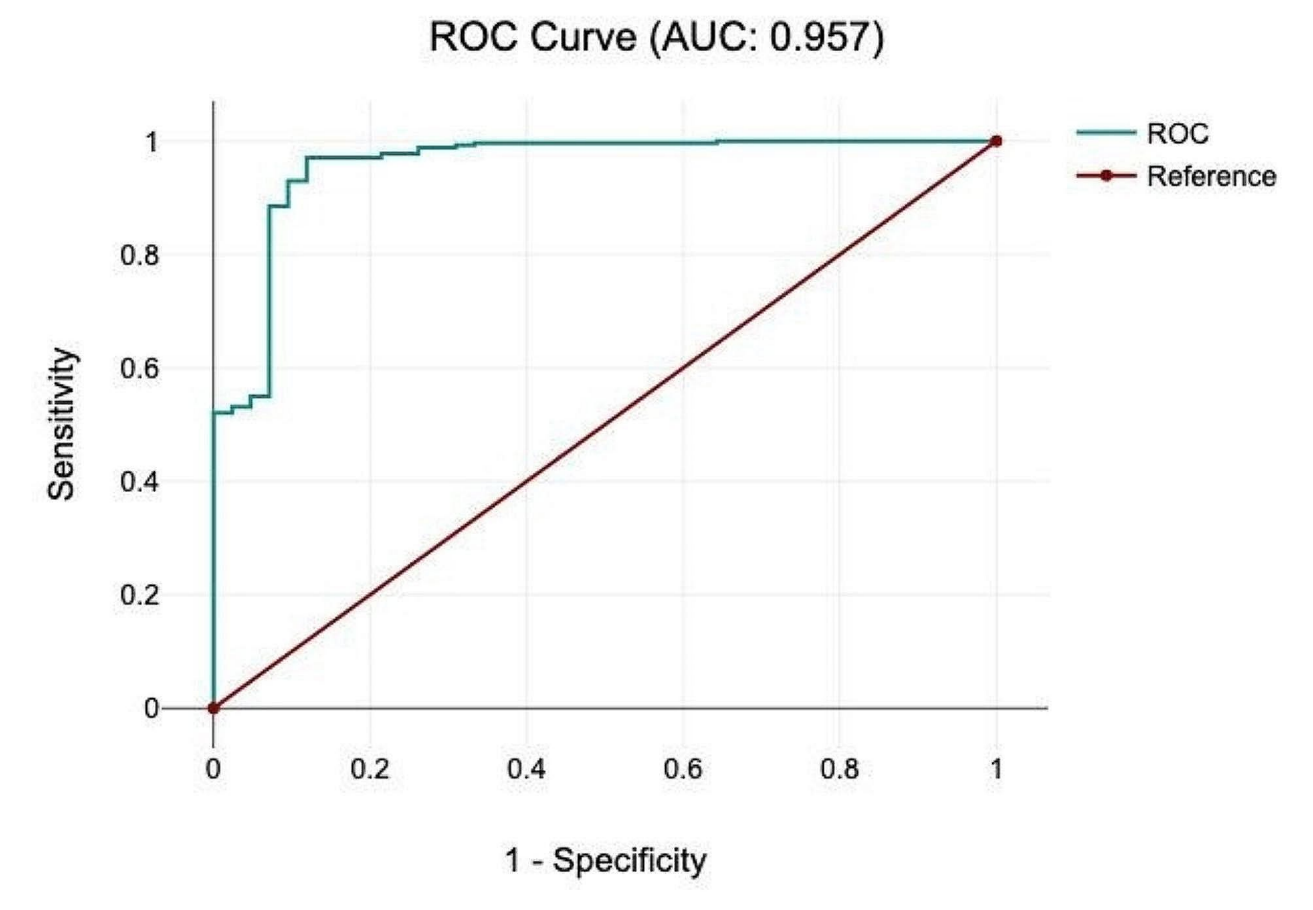
ROC-Curve

The area under the curve (AUC) score was 0.957 in the receiver operating characteristic (ROC) curve, which is an excellent prediction for mechanical ventilation (Fig. 3).

The logistic regression analysis to examine the influence of age, male gender, prehospital vitals, and characteristics on arrival at ED, mechanical ventilation, and ISS on variable mortality (dependent) showed that the model as a whole was significant (Chi^2^ = 63.78, *p* < 0.001). In particular, lower prehospital systolic BP (*p* = 0.049), on arrival at ED lower systolic BP (*P* = 0.013), lower SPO2 (*p* = 0.048), lower body temperature (*p* = 0.022), mechanical ventilation (*p* = 0.033), and higher ISS (*p* = 0.03) were significantly associated with mortality (Table 4).

**Table 4:** Logistic regression model to examine the influence of age, gender, pre-hospital vitals, and characteristics on arrival at ED on mortality

	Coefficient B	Standard error	z	*p*	Odds Ratio	95% CI
**Constant**	-154.33	8955.89	0.02	0.986	0	0 - Infinity
**Age**	-0.08	0.05	1.66	0.097	0.92	0.84–1.01
**Male gender**	-36.7	8955.25	0	0.997	0	0 - Infinity
**Prehospital**						
SBP (mm Hg)	-0.06	0.03	1.97	0.049*	0.94	0.89–1
HR (beats/min)	-0.03	0.04	0.71	0.48	0.97	0.91–1.05
RR (rate/min)	0.16	0.23	0.71	0.479	1.18	0.75–1.85
**On arrival at ED**						
SBP (mm Hg)	0.09	0.04	2.48	0.013*	1.1	1.02–1.18
HR (beats/min)	-0.03	0.04	0.68	0.499	0.97	0.9–1.05
RR (rate/min)	0.35	0.24	1.49	0.136	1.42	0.9–2.26
SPO_2_	0.59	0.3	1.98	0.048*	1.8	1.01–3.22
Body Temp (^0^C)	3.18	1.39	2.28	0.022*	24.09	1.57–369.59
GCS	-0.08	0.16	0.5	0.616	0.92	0.67–1.26
Blood P^H^	3.7	11.37	0.33	0.745	40.44	0–194017836362.64
Base Excess	0.52	0.36	1.42	0.154	1.68	0.82–3.43
INR	-4.89	2.57	1.91	0.057	0.01	0–1.15
Blood transfusion in ED	-2.2	1.57	1.4	0.161	0.11	0.01–2.39
Mechanical Ventilation	-4.82	2.26	2.13	0.033*	0.01	0–0.67
ISS	-0.14	0.06	2.18	0.030*	0.87	0.77–0.99

*Statistically significant *p*-value

**Fig. 4:**
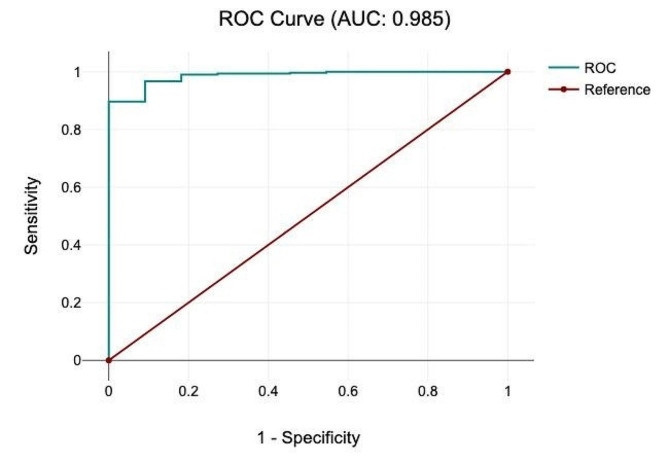
ROC curve

The area under the curve (AUC) score was 0.985 in the receiver operating characteristic (ROC) curve, which is an excellent prediction for mortality (Fig. 4).

## Discussion

The study investigated the important characteristics, trends, and seasonality of pedestrian traffic injuries presented to KSMC, a major trauma hub in Saudi Arabia. The study also investigated the impact of patients’ pre-hospital vitals and critical features upon presentation at the ED on the requirement for mechanical ventilation and in-hospital mortality.

Males constituted a disproportionate proportion of the injured, according to our findings. This finding is consistent with prior research conducted in Saudi Arabia and internationally [15, 16], which has concluded that males below the age of 40 are consistently more susceptible to RTCs, which encompass injuries to pedestrians. Based on our data, it is evident that males comprised an estimated 89.5% of the overall pedestrian injuries documented, which is an exceptionally high rate compared to all other demographic groups. This indicated a higher propensity for risk-taking behaviors among younger individuals, specifically males, including jaywalking, crossing roads without signaling for approaching traffic, and using mobile phones while crossing [17, 18]. Another contributing factor could be the average speed of vehicles on the road, which may be higher in areas such as commercial districts or entertainment venues. High-speed areas correlate with more severe injuries among pedestrians [19]. The over-representation of males in pedestrian injuries also suggests a gap in public policy. By identifying young males as a particularly vulnerable group, our study points to the urgent need for age- and gender-specific road safety measures. Interventions could include public awareness campaigns, putting more zebra crossings for pedestrian crossings, expanding cameras for road surveillance, fines for speeding, and changing urban planning that considers the specific behaviors and risk profiles associated with this demographic.

The majority of pedestrian injuries in our study involved non-Saudi patients, accounting for two-thirds of the cases. The expatriate community in the Kingdom accounted for one-third of the total population, amounting to at least 10 million individuals. Most of these expatriates were adult workers. Typically, they come from lower socioeconomic origins and rely on walking rather than using vehicles for their daily commute. This could lead to an increase in pedestrian injuries among this demographic [20].

Saudi Arabia, particularly Riyadh, experiences a desert climate with higher temperatures during the day than at night. People’s movements are often less frequent during the daytime than in the evening, resulting in a higher incidence of pedestrian injuries in the evening. This pattern was observed in two-thirds of the evening incidents in our study group. Moreover, sporadic sandstorms and evening visibility may also play a role. This could mean more people walking on the streets during the evening, comparatively less safe, and lower-visibility hours, thus contributing to the injury rate. Furthermore, the period between April and September corresponds to warmer months in Saudi Arabia. Decreased outdoor activity for the working-class group, especially expatriates, during the daytime may slightly decrease injury events. Saudi Arabia observes several festivals and national holidays during these months, which may correspond with increased population movement out of the city and contribute to fewer pedestrian injuries [21]. It’s worth noting that a similar seasonal trend has been observed in other regions, both globally and within the Middle East. However, our study’s observed pattern indicates possible local or cultural factors exacerbating the trend. Therefore, these findings indicate that targeted interventions and public safety campaigns could be especially beneficial during the peak months.

We found a significantly lower (13.28%) number of pedestrian injuries in 2020. The COVID-19 pandemic has compelled numerous governments to implement strict societal lockdowns and laws to mitigate the rapid transmission of the sickness. The initial occurrence of COVID-19 in Saudi Arabia was documented on March 2, 2020; subsequently, the number of cases rapidly escalated. In early March 2020, the Saudi Arabian government implemented measures to limit some activities, such as closing schools, universities, social events, and international flights. In addition, a stay-at-home mandate was implemented in Riyadh, the capital city of Saudi Arabia, on April 6, 2020 [13].

Our study also found that around 50% of the patients were transported to KSMC via Red Crescent ambulance. Since 1934, the Saudi Red Crescent Authority has been delivering prehospital care nationwide in Saudi Arabia. Upon activation, the Red Crescent ambulance and emergency medical staff promptly respond to the injury site and take victims to the closest hospital for immediate evaluation and resuscitation. Conversely, government ambulances facilitate the transportation of patients between hospitals, whereas private ambulances are responsible for transporting patients from private hospitals [22].

A key observation in our study was the high frequency of injuries in the lower extremities, which constituted 27.42% of all reported cases. This finding aligns with existing literature, often highlighting that the lower extremities—legs, knees, and feet—are the most vulnerable body parts in pedestrian traffic crashes [23, 24]. These injuries often range from fractures and dislocations to more severe forms like compound fractures, which can lead to long-term disability and impairment. The high incidence of lower extremity injuries may be attributed to the point of impact during a collision. In most pedestrian-vehicle encounters, a car’s front bumper and hood likely contact a pedestrian’s lower body, causing the observed injuries. The severity of such injuries often leads to prolonged hospital stays and, in many cases, disabilities. Consequently, the economic burden of treatment and rehabilitation for these injuries can be substantial, underscoring the necessity for preventive measures like traffic calming techniques and pedestrian safety barriers.

Head injuries followed closely, making up 21.37% of the cases in our study. This is particularly concerning given the high likelihood of these injuries leading to severe outcomes, including traumatic brain injuries (TBIs), comas, and even death. TBIs are often life-threatening and necessitate specialized care, including constant monitoring and, sometimes, surgical interventions. Our findings highlight the need for immediate and comprehensive trauma care protocols for patients presenting with head injuries, in line with global best practices. The prominence of head injuries in our study also corroborates existing literature, which notes that head and brain injuries often account for the most severe cases and are significant contributors to the high mortality rates associated with pedestrian injuries [24]. The fact that head injuries are the second most common type of injury but contribute significantly to the mortality rate indicates a serious public health concern. This also raises questions about the protective measures currently in place. For example, the absence of, or improperly designed, pedestrian crosswalks and signals may contribute to the risk of more severe injuries, such as those to the head. In addition to physical injuries, the psychological impact on victims should not be overlooked. The experience of a traumatic event like an RTC can lead to post-traumatic stress disorder (PTSD), anxiety, and depression, complicating the recovery process and requiring additional mental health services [25].

An injured patient is more prone to bleeding, causing hypotension, altered level of consciousness, requiring more blood transfusion, and more prone to have higher ISS. Such patients are also more vulnerable to maintaining airways and often require a definitive airway and mechanical ventilation. Our study observed that these variables were significantly correlated with mechanical ventilation and mortality. Additionally, severely injured bleeding patients suffer from lower oxygen saturation at the tissue level due to airway, breathing, or circulatory dysfunction. They also become more hypothermic, initiate a lethal cascade, and worsen outcomes, as observed as a significant predictor for mortality in our study [26]. Comparable findings were observed in the analysis of risk factors pertaining to hospital mortality among pedestrian-injured patients at a Level-I trauma center in Southern Iran. Specifically, the results indicated that hemoglobin level, GCS score, and ISS were independent determinants of hospital mortality in this population [27].

The mortality rate in our cohort was less than 5%, keeping similarities reported internationally [28]. This may be due to recent trauma system development in Saudi Arabia, improved prehospital care, a state-of-the-art ED facility, a dedicated trauma team, a highly specialized trauma surgeon, the availability of subspecialties, and the sophisticated ICU services at KSMC [29]. Although mortality depends on many other factors, including the severity of injuries, especially devastating head injuries, an improved trauma system can definitely save potentially salvageable deaths [29].

There were several limitations to this study. First, the study relies solely on registry-based hospital admission data. Consequently, pre-hospital mortality is not included in our analysis, potentially underestimating the true mortality rate and severity of pedestrian injuries in the population. Second, this study was conducted in a single major trauma center in Riyadh, which might not represent other regions in Saudi Arabia, especially rural or less-developed areas. Third, our study did not delve into the socioeconomic background of the injured pedestrians, which could provide additional layers of understanding regarding risk factors and outcomes. Finally, the nature of hospital-based data collection may introduce reporting bias, particularly in capturing less severe injuries that do not necessitate hospital admission but still have significant personal and public health implications.

Future studies should investigate community-based or national databases to provide a more holistic view, including pre-hospital deaths and injuries treated outside major trauma centers. To better understand the trends and patterns over time, longitudinal studies could offer deeper insights into the effects of policy changes or interventions. Incorporating socioeconomic data could offer a multifaceted understanding of the issue, identifying whether income, education level, or other social determinants influence the risk and outcomes of pedestrian injuries. The study’s findings about the specific age groups at risk should guide targeted public awareness campaigns to educate these populations about pedestrian safety. Future studies should also consider assessing the role of environmental factors like road design, signage, and traffic calming measures, especially in identified high-risk zones. Future research should also employ more complex statistical models that could further adjust for potential confounders, providing more robust findings. Comparing the trends and outcomes of pedestrian injuries in Saudi Arabia with other countries could offer valuable international perspectives and lessons.

## Conclusion

The present study comprehensively analyzes pedestrian injuries at a major trauma center in Saudi Arabia. The demographic and clinical characteristics, particularly the disproportionate representation of males and non-Saudi nationals and the timing of injury events, offer valuable insights into the population most at risk. The severity and complexity of injuries underline a significant healthcare burden, both in terms of immediate medical attention and long-term care. This study’s findings support the growing need for urgent preventive measures. These measures could include public campaigns to educate pedestrians and infrastructure improvements to make roads safer. The alarming trends identified in this study make a strong case for action from healthcare providers, policymakers, and community leaders.

## Data Availability

The datasets used during the current study are available from the corresponding author at reasonable request and with permission from the IRB, KSMC, Riyadh, Saudi Arabia.

## Abbreviations

AUC: Area Under the Curve
CI: Confidence Intervals
DAMA: Discharged Against Medical Advice
ED: Emergency Department
GCS: Glasgow Coma Scale
HR: Heart Rate
ICU: Intensive Care Unit
IQR: Interquartile Range
ISS: Injury Severity Score
KSMC: King Saud Medical City
ROC: Receiver Operating Characteristic
RR: Respiratory Rate
RTC: Road Traffic Crash
SBP: Systolic Blood Pressure
SD: Standard Deviation
STAR: Saudi TraumA Registry
TBI: Traumatic Brain Injury
WHO: World Health Organization
